# Conceptual Behaviour Underpinning the Occurrence of Nonfaecal Matter in Faecal Sludge in Some Urban Communities, Ghana

**DOI:** 10.1155/2021/2672491

**Published:** 2021-05-10

**Authors:** Ama Mbeaba Quarshie, Samuel Fosu Gyasi, Francis Atta Kuranchie, Esi Awuah, Eugene Darteh

**Affiliations:** ^1^Department of Civil and Environmental Engineering, School of Engineering, University of Energy and Natural Resources, Sunyani, Ghana; ^2^Department of Basic and Applied Biology, School of Sciences, University of Energy and Natural Resources, Sunyani, Ghana; ^3^Department of Civil Engineering, Kwame Nkrumah University of Science and Technology, Kumasi, Ghana; ^4^Department of Population and Health, University of Cape Coast, Cape Coast, Ghana

## Abstract

Faecal sludge (FS) management is pertinent to the achievement of sustainable development goal 6.2 around the world; yet it is constrained by urbanisation challenges, waste management complexities, and defective attitudes. These deny communities of the plausible supply of resources from FS. This paper assesses the perception underpinning the occurrence of nonfaecal matter in FS in Ghana. Primary data were obtained from 400 respondents in four communities in Brong Ahafo and Greater Accra Regions of Ghana, using a structured questionnaire. Data were analysed by using STATA software version 15. Chi-square test and multiple logistic regression were conducted on all independent variables and statistical significance was accepted at *p* < 0.05. The study identified the following as the most perceived frequently disposed nonfaecal matter into FS: sanitary pads and diapers (38.5%), fabrics/rags (23.2%), toilet rolls (20.8%), razor/shaving sticks (10.3%), and others (7.2%). Gender, state of toilet facility (roof or unroofed), presence of container for collecting other types of waste in the toilet room, and state of container in toilet room either covered or uncovered were the factors found to be significantly associated with the disposal of solid waste (SW) into FS at 95% confidence level. The fear of exposing used sanitary materials for rituals, the use of fabric as an alternative to toilet rolls, and the desire to conceal aborted pregnancies from the public were some of the reasons alluded to the disposal acts. Education and awareness campaigns on proper SW disposal practices, appropriate use of toilet facilities, and the resource potentials of FS were found to be the best way forward to discourage indiscriminate disposal of SW into FS.

## 1. Introduction

Indiscriminate disposal of solid waste (SW) has become a global challenge, particularly in most developing countries. Vacant lands and roadsides in and around rivers as well as trenches have been used as disposal sites and also for the burning waste [1–4], thus deteriorating environmental sanitation situations in most developing countries [5]. The indiscriminate manner of SW disposal has become a canker not only for developing countries but also for a number of developed ones as well. Waste management in “First Nations” communities such as Garden Hill and Wasagamack in Canada has also become a problem, though Canada is ranked 24th among 178 countries on the Environmental Performance Index 2 [3]. Nonetheless, indiscriminate dumping of SW is more prevalent in developing countries than developed ones. Aziale and Asafo-Adjei [6] estimated that about 80% of total SW volumes were disposed indiscriminately in African countries.

In Ghana, a number of efforts aimed at solving sanitation problems have been instituted; one of these is the institution of “National Sanitation Days”; yet this has not yielded much result as they were exclusive of the adjustment of attitudes of people. The need for a paradigm shift from the old approaches of addressing sanitation problem is relevant now, since the menace is gradually being detected in unthinkable confined places such as the toilet facilities/faecal sludge (FS). Already, the task of managing high volumes of FS in populated urban areas of most African countries has been unnerving due to urbanisation challenges such as poor living conditions and inadequate FS treatment facilities [7].

Nonbiodegradable SW in FS is now a common occurrence in pit latrines/toilet facilities, thus worsening the prevailing problem of FS management. According to some researchers, on-site sanitation facilities in urban slum settlements are mainly accumulating nonfaecal material as they mostly use these areas as disposal sites for their SW [5, 8]. This assertion resonates with Zziwa et al., [9] who explained that the design of pit latrines allows some communities dwellers to use them as disposal points for their SW as they evaluate that 15% of pit latrine content was found to consist of paper, textiles, and feminine waste among others in Uganda. Sponges, bones, wood, textiles, plant seeds, stones, plastics, and sand are usually found in FS extracted from on-site facilities [10]. During their field trials in South Africa, Bakare et al. [11] also observed a large amount of household SW in the FS of their study population. Gudda et al. [12] disclose that latrines that serve as reservoirs for FS and the environment are now areas where SW is purposefully or randomly disposed in Kenya. Mourad's [13] report on sewer blockage in Australia during the most recent outbreak of the COVID-19 pandemic resulting from the extensive cleaning with wet wipes, tissues, and paper towels due to the shortage of toilet rolls raises concern about people's indiscriminate disposal of SW into FS. In a most recent study of some communities in Accra, Ghana, an estimated 2.17 kg of SW per cesspit truck of FS (0.24 kg/m^3^) was identified [5].

There have been several attempts to explain the upsurge of this mode of disposal of domestic waste, resulting from poverty, low standards of living, high population, low level of environmental awareness, and inadequate management of environmental knowledge, among others [14], like all other forms of littering. Mwiinga [15] absolutely blames the rise of indiscriminate disposal of SW on defective attitudes, perceptions, and faulty evaluation of waste disposal issues. Some community dwellers have considered FS and SW as waste which are of no value at all; so, assume the practice of disposing SW into pit latrines which exclusively serve as containment for FS as an acceptable practice. As such, disposing other equally unwanted materials into them should be suitable. Yet, the effect of dumping SW into FS has been well documented to include the shortening of the fill-up rates of latrines [8, 9, 16], the frequent blockage of sewers, and thus dents' community sanitation.

The design specifications of the FS treatment plant highly hinge on FS variability and so must depend on the local characteristics of the FS to include the presence of SW in the sludge [17]. This aids appropriate and accurate design to resolve treatment challenges [18]. Quality of treatment is significantly affected by the screening efficiency of the plant [19]; thus, the presence of all kinds of SW in FS can render its treatment problematic as most treatment plants may not have design specifications to deal with these kinds of waste. The prevention of SW accumulation in FS is therefore paramount.

Although a plethora of studies are replete with either the accidental or purposeful disposal of SW in FS, studies meant to ascertain the reasons for this practice have not been fully explored. This study sought to assess the perception and knowledge base of the public in some selected communities in the Accra Metropolis and Sunyani Municipality of Ghana on the disposal of SW materials into FS. An understanding of the knowledge base of the public is apposite in developing appropriate policy for the mitigation of defective attitudes that fuel general SW disposal practices and by extension prevent the load of nonbiodegradable waste into FS. This ensures the availability and sustainable management of water and sanitation for all as indicated in SDG 6.

## 2. Materials and Methods

### 2.1. Study Area and Population

The study was conducted in two urban districts in Ghana, West Africa. These included the Accra Metropolitan and the Sunyani Municipal Areas. The study area for the Sunyani Municipality lies between latitude 7°06′–7°24′ north and longitude 2°12′–2°30′ west, while that of the Accra Metropolis lies within latitude 5°28′–5°40′ north and longitude 0°8′–0°16′ west. The vegetation around the Sunyani Municipality consists of Moist-Semi-Deciduous Forest Vegetation Zone with a population of 123,224 and dominant occupations as farming and trading; Accra Metropolis is located within the grassland vegetation zone of the country with a population size of 1,848,614 and noted for trading and fishing occupations.

### 2.2. Selection of Study Communities

The entire country was categorised into the Northern and Southern zones with each zone comprising 5 metropolitan/municipal areas. The Northern zone had Sunyani as the only municipality with the highest patronage of public toilet facility among the household population. All 5 municipalities/metropolis in the Southern zone had public toilets as the main toilet facility, with Accra being the metropolis with public toilet facility patronage concentrated in its urban areas only. These influenced the selection of Sunyani Municipality and Accra Metropolis as study areas. Due to data paucity on the community patronage of public toilet at the household level in the entire country to aid the selection of communities for the study, the environmental health executives of each study area were consulted for the characteristics of the communities within their respective jurisdictions based on the population densities, living standard, and sanitation challenges as prescribed by [14] to aid the purposive sample of two communities from each study area.

**Figure 1:**
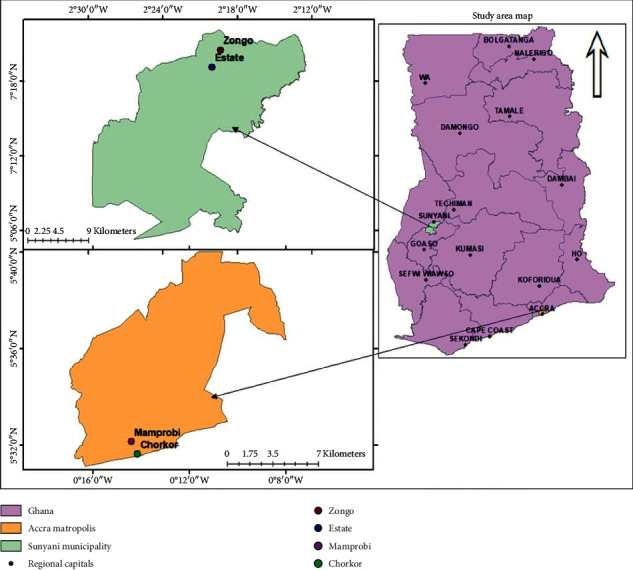
Study area map.

Two hundred respondents were then arbitrarily sampled from each study area through the simple random sampling method. The researcher selected two communities each; they were purposively sampled based on population densities, living standards, and sanitation challenges as prescribed by [14]; these factors are likely to influence the accumulation of SW on FS. The environmental health executives of the two areas for the study were consulted for the characteristics of the communities within their respective jurisdictions based on the above criteria. This informed the selection of the Zongo and Estate communities for the Sunyani Municipality and the Mamprobi and Chorkor communities for Accra Metropolis, respectively, as shown in Figure 1.

### 2.3. Study Design

The study adopted a community-based cross-sectional approach since the data gathered are a snapshot of what is happening in the total population at a point in time.

### 2.4. Sampling Method

The simple random sampling technique was employed; respondents were selected using a convenient sampling technique as they visit the toilet facility during the day. To calculate the sample size, the following formula was used: *n*=*Z*^2^*pq*/*d*^2^ [20], where *n* = the desired sample size, *z* = 95% confidence interval (standard value = 1.96), and *p* = the actual proportion of respondents who disposed of SW into FS. Since the actual proportion is unknown in literature, 50% (0.5 in probability terms) was used. A 5% nonresponse was allowed and the total sample size was estimated to be 400. Respondents in each of the four communities were selected based on proportionate to sample size technique until the total sample size of 400 was attained. Questionnaire was the main research tool used for the gathering of data. The questions focused on respondents' demographic data, household waste generation and disposal practices, nature of toilet facilities, toilet facility usage, opinion on SW in FS, and opinion and knowledge on environmental awareness and education. Opinion on the utilization of recycled products from FS was also assessed.

#### 2.4.1. Quality Assurance

Proper quality assurance procedures and precautions were taken to ensure the reliability of the questionnaire. Pretesting of the questionnaire was conducted to ascertain the reliability of the information gathered. The reliability of the information was confirmed by examining the individual test items with Cronbach's alpha. A Cronbach's alpha value of 0.78 was obtained which indicates an excellent internal consistency of the questionnaire. For the validation of the questions, two research fellows were contacted to review it and their comments were addressed to improve the reliability of the questionnaire before actual field data collection.

#### 2.4.2. Ethical Issues

Participants' confidentiality was guaranteed since their names were not included in the questionnaires. Also, the research did not refer to each individual name of participants in the manuscript and this assurance was also given to participants. A permission notice was attached to the questionnaires in clear and simple language for easy understanding and this was further explained clearly to all participants. Consent was received from all participants before the interview began. Finally, the University of Energy and Natural Resources gave approval for this research to proceed after due diligence consideration for all ethical issues.

### 2.5. Statistical Analysis

The questionnaire was entered into Microsoft Excel spreadsheet 2013; the data were cleaned and imported onto STATA statistical software version 15. Descriptive statistics such as frequencies, standard deviations, and 95% confidence intervals for all variables were obtained and the result was presented in the form of tables and charts. Bivariate analyses using Chi-square test statistics (*χ*^2^) were performed to test the associations between independent and dependent variables. Where one of the cell frequencies was more than 5, Fishers exact test was used. Multiple logistic regression was conducted on all independent variables and crude odds ratio (cOR) and adjusted odds ratio (AOR) was determined. A *p* < 0.05 was considered statistically significant.

## 3. Results

Table 1 is a summary of the demographic characteristics of the respondents. The results showed that 33% were between the ages of 20–29 years with a mean age of 29.5 ± 0.39 (95% Cl: 25.65–32.0). The majority (58%) were males. Most of the study respondents (54.3%) had attained basic education, 22% had secondary education, and 11.8% had no formal education. With regard to religious affiliation, 58.8% were of Christian faith and 36.3% were of Islamic faith. The analyses of the occupational backgrounds of the respondents revealed that a sizeable number (72%) of the entire population of respondents were employed and 28% were unemployed; those who were unemployed were either students or not engaging in any form of economic activities that generate income. Most respondents had a household size of at least 10 people representing 45%, 32% had an average household size of about 6–9 people, and 20% had 2–5 people.

**Figure 2:**
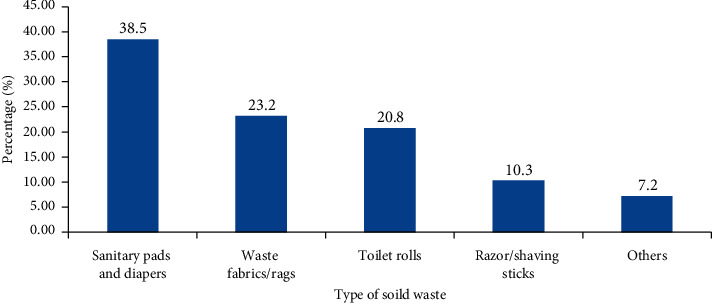
Type of solid waste (SW) respondents disposed into faecal sludge (FS).

The results showed that, out of the 400 respondents, 242 (60.5%) had ever disposed of SW into FS. However, the study found that 48 out of the 242 asserted that it was accidental. This indicates that the proportion of respondents who had ever disposed of SW into FS was 194, representing 48.5% of the total population sampled. The most frequently disposed SW reported was sanitary pads and diapers (38.5%), fabrics/rags (23.2%), toilet rolls (20.8), razors/shaving sticks (10.3%), and others such as condoms (7.2%) (Figure 2).

Explaining why fabrics such as cloth, pants, and handkerchiefs are found in FS, 52.5% of the respondents indicated that fabrics are normally used in the absence of tissues or toilet rolls for cleansing, 28.5% of respondents were of the opinion that the presence of fabrics in FS can be linked to their usage in place of sanitary pads which are mostly disposed into the FS after cleansing, and 19.0% respondents inferred that fabrics were sometimes accidentally dropped in FS.

A significant number of respondents forming 47.2% explained that sanitary pads are found in the FS because those who dispose them into the FS want to prevent them from being used for rituals as may be the case when they are rather disposed together with domestic waste but to 39.2% respondents, it is simply a taboo for menstrual materials like sanitary pads to be disposed in such a manner that they will be exposed to the sight of the public. 13.6% asserted that sanitary pads are found in the FS because toilet rooms are usually used by ladies as convenient places to change their pads; thus, the likelihood of disposing them into the FS is high.

Respondent's view on whether or not diapers are disposed in the toilets because they contained faecal matter already revealed that 69% of the entire respondents agree on the claim while 31% did not agree. 65% of the respondents also acknowledged that razors, piercing instruments, and other types of hazardous waste are dumped into the FS to prevent exposing them to children but 35% disagreed.

Concerning the generation and storage of waste, the results showed that 48.2% of the total respondents store their SW in public waste bins, while the rest either bury or burn them in backyards. On the conceptual estimation of the percentage of plastics in the overall SW generated, 52.5% indicated that plastics/rubbers were the major composition of their domestic waste and ranged from 25 to 40%, thus posing a significant risk of plastic waste accumulation in the FS. A significant proportion of the respondents (65%) indicated that they sometimes find rubbish around waste collection bins, whereas 35% of respondents asserted that they did not find waste scattered around waste bins.

On the reasons attributed to why waste is found around waste bins, 42.7% opined that the capacity/size of the waste bins could be a reason why SW is scattered around the bins while the remaining 57.3% attributed it to mere indiscriminate disposal attitude of people. The proximity of the dumpsites from respondents' houses was assessed to investigate if that could be the reason for the disposal of SW in FS and 36% of the total respondents asserted that the distance from their houses to the waste dump sites is at least 500 m, while 64% reported that the distance between their houses and the waste dump site was less than 500 m.

Approximately 91% (91.4%) indicated that they have toilet facilities in their houses/suburbs. Among the types of toilet facilities that respondents use as a place of convenience, 68.2% of total respondents disclosed that they use the public toilet. The majority (80%) had their toilet facilities roofed but 41.2% of total respondents said that they have no doors on the entrance of their toilet facilities which gives a high exposure of the FS to SW accumulation. 42% of the entire respondents responded that their domestic waste collection bins were sited close to toilet facilities making the toilet facilities more prone to SW accumulation.

The study found gender to be the demographic factor that was significantly associated with the disposal of SW into FS at 95% confidence level (*p* < 0.05). Females were approximately 9 times more likely to dispose SW into FS compared to their male counterparts (AOR = 8.7; 95% CI = 0.037–0.849, *p*=0.037). Other variables such as the state of toilet facility (roof or unroofed); the presence of a waste bin in the toilet room; state of container in toilet room either covered or uncovered were the factors found to be significantly associated with the disposal of SW into FS at 95% confidence level (Table 2).

Adjusting for all other factors, respondents who had their toilet facility roofed had 32% reduced odds of disposing SW into FS compared to those who had their toilet facility unroofed (AOR = 0.68; 95% CI = 0.207–0.863, *p* < 0.037). Respondents who had containers in the toilet room for collecting other types of SW had 48% reduced odds of disposing SW into FS compared to those who had no containers (AOR = 0.52; 95% CI = 0.379–0.728, *p*=0.025). Respondents who shared toilet facility with other people were approximately 5 times more likely to dispose SW into FS compared to those who do not share their toilet facility with other people (AOR = 5.20; 95% CI = 1.67475–11.328, *p*=0.021). Respondents who have covered bins in their toilet rooms had 68% reduced odds of disposing SW into FS compared to those who had their containers uncovered (AOR = 0.32; 95% CI = 0.178–0.628, *p*=0.002) (Table 2).

Respondent's perception on whether or not they find waste collection bins in the toilet rooms showed a significantly high percentage of the entire population (81.7%) confirming they do, with only 18.3% disclosing that waste bins are not normally found in the toilet rooms. Seventy percent (70%) of the respondents asserted that most of the waste bins in their toilet rooms are not covered. Approximately 78% of respondents mentioned that they had waste bins in the toilet room and 22% did not. 20.5% of the respondents asserted that they dispose their sanitary waste into the FS in the absence of the waste bins in the toilet rooms, but 2% of the respondents claimed that they leave their used cleansing material on the floor of the toilet room when the waste bins are not available. These suggest that the majority of the respondents opt for other means of disposing their cleansing materials in the absence of waste bins in the toilet rooms. Ironically, most respondents (57.2%) confirmed that they find sanitary materials scattered on the floor of the toilet rooms.

Most people do not put in any effort to tidy up the toilet rooms when dirty; this was confirmed by 49% of respondents, while 51% of the total respondents said they make effort from their own volition to tidy up the toilet rooms when they find them untidy. There is a clear indication that users of most toilet facilities feel reluctant to clean up the toilet rooms when untidy. 59.7% of respondents said that burning, burying, and throwing their cleansing materials away in bushes and nearby pits are others means they dispose outside the toilet facility; 31.2% respondents disclosed that they dispose the sanitary waste into waste bins found in the toilet rooms, while 9.1% of the overall respondents for the study disclosed that they dispose waste into the toilet. It is quite evident that the majority of the respondents prefer to dispose the gathered waste from the toilet rooms outside the toilet premises.

**Figure 3:**
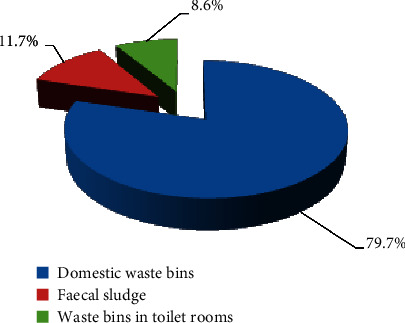
Disposal of used diapers by females.

On the issue of whether or not domestic waste is combined with waste collected from the toilet before final disposal, most respondents (46%) disclosed that they do not combine sanitary/cleaning waste with the household waste; however, 54% of total respondents said that they usually do combine the waste collected from toilet with other domestic waste materials before they dispose them. Views on where used diapers are normally disposed showed that 79.7% mostly women asserting that they usually prefer to put used diapers in polythene bags and dispose them into domestic waste/rubbish bins wrapped, whereas 11.7% indicated that they disposed it directly into faeces/toilet, and 8.6% respondents disclosed that they dispose used diapers in waste bins placed inside the toilet rooms (Figure 3).

A significant number of respondents (88%) disclosed that razors/piercing instruments are disposed into household/domestic waste bins after usage, but 8.7% affirmed that those types of waste are usually disposed directly into the FS when used at the toilet facility, while 3.3% indicated that they dispose it into waste bins positioned in toilet rooms.

On respondents' reasons for the disposal of domestic waste into FS, a number of respondents representing 35.5% disclosed that people's action was due to their flawed perception. They perceive FS/toilet as suitable medium for disposing domestic waste because it is also waste. 14.5% of the overall respondents revealed that people dispose waste into FS due to their ignorance on the proper means of waste disposal but 12.7% of the respondents said that such disposals are accidental, while 4.7% disagreed and indicated that they are done deliberately. 22.7% of the respondents explained that such disposal act is due to the absence of waste bins in the toilet rooms while 9.9% of respondents justified the act and explained that FS facilitates the decomposition of the waste they put into it.

The study findings showed that education on indiscriminate disposal of SW into FS is inadequate as disclosed by the majority of the respondents (77.5%); yet 22.5% of the respondents indicated that there has been some form of education against the disposal of SW in FS in their community. The opinions of the respondents on how indiscriminate disposal of waste into the FS could be prevented revealing education as the major means by which the act can be prevented as this was prescribed by 39% but 25.2% of the overall respondents rather suggested that the provision of enough waste bins in the toilet rooms will help avert the accumulation of SW into FS. 17.2% of the respondents prescribed punishment as the best option to prevent the act. Other respondents, 18.6%, supported the view that keeping toilet facilities clean will help resolve the problem of indiscriminate disposal of SW into FS.

Respondents' knowledge of any challenges associated with the indiscriminate SW disposal into FS brought to the fore its tendencies of increasing the contraction of infectious diseases in the community. This was opined by 33.7% of the respondents but to 24.5%; the act could lead to the clogging of the septic systems, while 17% of respondents believed that it will rather lead to an unattractive and unhygienic state of the toilet facilities. Nonetheless, 13.5% of respondents alleged that it would result in the increasing volumes of the sewage system at an alarming rate; 11.3% of respondents indicated that indiscriminate disposal of SW into the FS attracts opportunistic animals to the toilet rooms.

About 70% of respondents suggested that the Metropolitan, Municipal, and District Authorities (MMDAs) are responsible for educating the public against the disposal of SW into FS, but 21% rather suggested that users of the facilities should be educated by their peers, while about 9% pointed that the nongovernmental organisations should be responsible for that.

Respondents' perceptions on the utilization preferences of products made from processed FS were sought; 88% of the entire respondents declared that they would prefer to use recycled products from FS but 12% others indicated that they will not use products processed from FS, whereas the majority (92%) of the respondents could not give any reason why they think people would not prefer to use products recycled from FS, 8% attributed people's failure to use recycled products from FS to the perceived smell that the products might give off. Some 57.5% of the entire respondents disclosed that people would opt to use biogas, while 22.2% of respond others identified compost as the preferred choice of people for the product made from FS. About 9.2% rather indicated that people will prefer to use Briquette but some 11.2% of respondents acknowledged that they would desire to use bricks made from FS to build.

## 4. Discussion

Responses on the plastic waste compositions of domestic waste in the selected communities for the study were found to range between 21 and 40% of their total household waste; this finding is consistent with the findings of many researches such as one in Kenya that revealed polythene/plastic materials as the dominant material among the total SW generated in the majority of households [21]. High percentage of plastic proportion of domestic waste can pose high exposure to waste accumulation in FS. Although waste bins were mainly used to store domestic waste, their sizes were perceived by the respondents to be too small to contain the volumes of waste generated and as a result, respondent mostly found waste scattered around bins [4]. An estimated distance of more than 500 metres between the waste dump site and households does not only project a high risk of deposition of domestic waste along the path to the dump sites but into latrines and other obscure places closer to them as most respondents for this study estimated the distance from their houses to the waste dump sites as far.

Although most respondents said their toilet facilities are roofed, it was observed that respondents who had their toilet facility roofed had 32% reduced odds of having SW disposed into FS compared to those that had their toilet facility unroofed. This finding agrees with that of [22] clarifying that the absence of roofing on toilet facilities encourages other SW in the waste stream to be blown by wind into the FS. However, other researchers also opined that waste disposed into the FS is as a result of the toilet rooms without bins inside or lids covering the bins if they were provided and not necessarily the absence of roofing. These bins without lids in the toilet facilities also present a high risk of deposition of SW into the FS and therefore pose a significant nuisance which discourages the use of toilet facilities, especially when shared with others [4, 22, 23].

Toilet rooms without waste bins increase the chances of SW accumulation in FS. Respondents who had containers in their toilet rooms had 48% reduced odds of disposing SW into FS compared to those who had no containers. Although there was a significant difference in the patronage of public toilets/latrines without bins among community members as confirmed by Obeng et al. [23], Ghana has about 85% of households relying on communal latrines and open defaecation. It also had the highest population that depends on shared toilet facilities. Most toilet rooms were found unclean [24] because users do not take up the duty to clean up [9] and therefore pose a high risk of accumulation of sanitary waste into FS. They rather expect city authorities to be responsible for cleaning since they alleged paying for the maintenance of the facility as enshrined in the pay-per-visit tariff policy. According to Oduro-Kwarteng and Pieter van Dijk [2], users will be unwilling to pay for using the facility when they presume that the services rendered are of poor quality.

The act of burying, burning, and dumping of waste into bushes, pits, or domestic waste bins among community members is popular modes of disposing sanitary waste other than the normal practice of disposing them into bins provided or supposed to be available inside the toilet rooms. Sanitary pads and used diapers were the most frequently disposed waste into FS [24] and pose a significant risk of increasing SW accumulation in FS, in addition to are fabrics, razors, toilet rolls, and foetus. Other reasons that could be attributed to the disposal of solid waste into FS other than the ones mentioned by Murphy [24] are the perception that children and scavengers were safe when razors, syringes, and others are deposited into FS instead of bins.

Confirming the above accession, Bakare [8] explained that materials that cannot be easily disposed by burning find their way into FS. Also in line with this is the observation that, as part of the protocol for the prevention of the spread of the recent COVID-19 pandemic, the public was advised to wash their hands thoroughly and dispose the tissue into bins but people preferred to dispose them into toilets and has caused blockage of some sewers especially in Australia as disclosed by Mourad [13]. He added that the toilet rolls crises resulting from their extensive use in the period of the pandemic in Australia also saw wipes, tissues, and towels viewed flushable but not all such materials have similar properties like the toilet rolls to be flushed into toilet facilities since they do not easily break down and therefore block sewers. He further explained that the disposal of SW into toilet facilities was as a result of behavioural induced factors and so advised that make-up wipes, cleaning wipes, cigarette butts, cotton buds, nappies, sanitary pads, condoms, dental floss, left-over medication, and hair should never be flushed down the toilet.

Undeniably, the cultural orientation of Ghanaians detests women who expose their used sanitary pads to the public so they find the FS as the convenient place for disposing them. Again, since toilet rooms have become the main place females change up their sanitary pads, any other materials used in the absence of the pads such as fabrics are also commonly disposed into it if they were not rather used to wrap aborted foetus before their intentional disposal into the FS.

It was also evident in the study that though the male respondents were in the majority, mostly youthful and so may be sexually active, they failed to disclose their perception on where used condoms are usually disposed. This was not surprising since again, it is abominable in the African context to discuss sex-related issues in public so respondents naturally shied away from any discourse that related to their sexuality; yet in a study by Ahmed et al. [5], used condoms constituted 3.7% of the total SW found in FS dislodged for treatment in Accra. Again, where waste is normally not segregated prior to disposal as it occurs in most developing countries, the act of wrapping almost everything including waste in polythene bags [21] before disposal accounts for the high occurrence of polythene strands in FS [9, 11, 25, 26]. The perceived erroneous justification for the disposal of diapers into FS is fuelled by the public's perception that as waste begets waste, faecal matter also begets faecal matter, so since used diapers usually contained faecal matter, it is usual to dispose diapers into FS. Thus far, the presence of nonfaecal materials in the FS is worrisome as according to [24, 27], it takes about a year for them to decay.

Some accidentally disposed items into FS include monies, mobile phones, rings, spectacles, keys, and sponges, among others. The intentional disposal of SW into FS has been predicted to be fuelled by the arbitrary disposal of waste into the environment 8, 25. Community dump sites estimated to be situated “far” (i.e., 500 metres or more) from most households also encourage indiscriminate disposal of waste in their surroundings, as people are usually reluctant to trek long distances to dispose of their waste [28]. Ironically, it was observed during the study that most community waste collection dumpsites were located closer to the public/community toilets and could also account for the presence of SW in FS.

The disposal of SW into FS causes untidiness and intense odour of the toilet facilities, thus discouraging people from using the facility. This can encourage open defaecation and worsen community sanitation as predicted by Obeng et al., [23] to include among the above the distance to the nearest latrine and the pay-per-visit tariff. Respondents also identified the possible tendencies of the contraction of diseases from the usage of toilet facilities laden with SW as one of the challenges SW poses when disposed into FS. They also submit that the cleanliness of the toilet facilities is as important as the safety of those places, not necessarily because of the ease cleanliness it may provide, but also because the protection it will give against diseases. Nonetheless, the clogging of sewerage systems as opined by Ahmed et al., [5] and Mourad S. [13] can as well be a challenge that encourages the practice of open defaecation.

Education on the prevention of SW disposal into FS in most communities in Ghana has been predicted to be inadequate, thus resulting in the frequent occurrence of SW materials in FS [9, 11]. Mwiinga [15] suggested that educating the public on the management of domestic waste and its effects on the environment can help curb the build-up of waste at undesignated areas while Obeng et al. [23] also emphasized the need for social interventions to make latrines more hygienic. The MMDAs were prescribed as the agencies responsible for embarking on these educational intervention programs towards the prevention of SW disposal into FS.

The knowledge base of most communities on the use of products from FS is only limited to biogas as is indicative of people's preference for biogas. In line with this, Asumadu-Sarkodie and Owusu [29] establish that biogas has substituted the use of biomass for cooking and heating purposes in Ghana and has resulted in less demand for biomass. This shift is what gives much popularity to biogas than all the other recycled products from FS. Diener et al. [30] supported this claim and reiterated that biogas is the most commonly used product from FS among Ghanaian communities; this is an indication that most people are not aware of the other recycled products from FS. Recognising this fact, Kuwornu et al. [31] suggested the need for such resources to be tactfully introduced to consumers through the explanation of its production processes and benefits in order to aid better understanding of these resources for them to be appreciated.

## 5. Conclusion

The shared responses in all four communities in the Accra Metropolis and Sunyani Municipality revealed that people mostly relied on community latrines which in most cases were untidy. Efforts to keep them clean were left to city/community authorities since people held the view that they pay for the usage of the facility and so the maintenance of the facilities must be the responsibility of the authorities. Sanitary pads, diapers, fabric, toilet roll, and condoms, among others, were alleged to be the kinds of waste mainly disposed into FS. Gender, state of toilet facility (roof or unroofed), presence of waste bins in the toilet room, and state of container in toilet room either covered or uncovered were the factors found to be significantly associated with the disposed of SW into FS. The fear of exposing one's used sanitary materials for rituals and the use of fabric as an alternative to toilet rolls were some of the reasons alluded for the inconsiderate disposal acts. Although disposal into FS may sometimes be accidental or spontaneous as in the case of the COVID-19 pandemic, this not only results from the reasons above but also hinges on the erroneous perception that FS is itself waste (unwanted) and so it is usual to dispose unwanted items into it.

Education on the proper use of the toilet facility and against the disposal of SW (cleansing and other materials) into FS is lacking and brings about FS processing challenges when potential resources are being recovered from it. A well-structured educational intervention program is therefore recommended to educate people against their general indiscriminate SW disposal practices and specifically against the disposal of SW into FS; also, the WASH programme should include the campaign against the indiscriminate disposal of SW into FS to improve community sanitation, enhance FS management processes, and result in the maximum recovery of the potentials communities can gain from this limitless supply of resource.

Waste management should be introduced in school curricula as a course of study to help develop an appropriate conceptual framework on FS. The various stakeholders therefore have a stake in facilitating change in perceptions, attitudes, and practices of community members towards the disposal of SW into FS. Further research can be conducted to examine the perception that informs the siting of most community dump site closer to community latrines.

## Figures and Tables

**Table 1:** Sociodemographic characteristics of respondents (*N* = 400).

Variable	Frequency	Percentage
*Age (years)*		
<19	31	7.8
20–29	132	33.0
30–39	92	23.0
40–49	75	18.8
50–59	48	12.0
≥60	22	6.2

Mean ± SD 29.5 ± 0.39 (95% Cl:25.65–32.0)		
*Sex*		
Male	232	58.0
Female	168	42.0

*Educational level*		
None	47	11.8
Basic	217	54.3
Secondary	88	22.0
Tertiary	48	11.9

*Religion*		
Islam	145	36.3
Christianity	235	58.8
Traditionalist	18	4.5
Others	2	0.4

*Occupation*		
Unemployed	112	28.0
Employed	288	72.0

*Household size*		
1	12	3.0
2–5	80	20.0
6–9	128	32.0
≥10	180	45.0

SD: standard deviation. CI: confidence interval. Source: field work, 2019.

**Table 2:** Multiple Logistics regression of the factors associated disposal of solid waste (SW) into faecal sludge (FS).

Variables	Disposal of SW into FS, yes (%)	No (%)	COR (95% CI)	*p*-value	AOR (95% CI)	*p*-value
*Gender*						
Male	62 (32.0)	170 (82.5)	1.0 (ref)		1.0 (ref)	
Female	132 (68.0)	36 (17.5)	9.2 (4.12–14.27)	0.018^*∗*^	8.7 (5.49–15.28)	0.002

*Share toilet facility with other people*						
No	46 (23.7)	6 (97.1)	1.0 (ref)		1.0 (ref)	
Yes	148 (76.3)	200 (2.9)	4.5 (1.493–3.468)	0.032^*∗*^	5.2 (1.674–11.328)	0.021^*∗*^

*State of toilet facility*						
Unroofed	74 (38.1)	6 (2.9)	1.0 (ref)		1.0 (ref)	
Roofed	120 (61.9)	200 (97.1)	0.51 (0.164–0.709)	0.015^*∗*^	0.68 (0.207–0.863)	0.037^*∗*^

*Presence of container at toilet room*						
No	58 (8.4)	30 (21.1)	1.0 (ref)		1.0 (ref)	
Yes	136 (19.2)	176 (23.9)	0.40 (0.132–0.921)	0.04^*∗*^	0.52 (0.379–0.728)	0.025^*∗*^

*State of container in toilet room*						
Uncovered	154 (79.4)	126 (61.2)	1.0 (ref)		1.0 (ref)	
Covered	40 (20.6)	80 (38.8)	0.18 (0.028–0.545)	0.001^*∗*^	0.32 (0.178–0.628)	0.002^*∗*^

^*∗*^Significant (*p* < 0.05); ref: reference category, cOR: crude odds ratio, and AOR: adjusted odds ratio.

## Data Availability

All data obtained from the survey have been included as tables and figures in this research.
